# Exogenous pesticide poisoning in the state of Espírito Santo, Brazil, 2007-2016: spatial distribution and temporal trend in the incidence rate and case fatality ratio of notified cases

**DOI:** 10.1590/S2237-96222022000200008

**Published:** 2022-07-08

**Authors:** Karla Patrício Carvalho, Rafael Belo Corassa, Glenda Blaser Petarli, Monica Cattafesta, Eliana Zandonade, Luciane Bresciani Salaroli

**Affiliations:** 1 Programa de Pós-Graduação em Saúde Coletiva, Universidade Federal do Espírito Santo, Vitória, ES, Brazil

**Keywords:** Agrochemicals, Poisoning, Health Information Systems, Spatio-Temporal Analysis, Mortality

## Abstract

**Objective:**

To describe the spatial distribution of pesticide poisoning cases and analyze the temporal trend in the incidence rate and case fatality ratio of this event in the state of Espírito Santo, Brazil.

**Methods:**

This was a time series study of confirmed cases of pesticide poisoning registered on the Notifiable Health Conditions Information System (SINAN) between 2007 and 2016. Incidence rates were presented in thematic maps. Scatter plots were used to show the incidence and case fatality ratio of cases.

**Results:**

There was an increasing incidence rate of poisoning in the period regarding the total number of cases, by sex and age group - except for those aged 70 years and over. There was a decreasing case fatality ratio until 2012, with a subsequent increase. Eight municipalities presented the highest notification rates (30 to 46 new cases/100,000 inhabitants).

**Conclusion:**

There was an increase in the incidence of notifications in the period. There was a decrease in case fatality ratio until 2012, subsequently followed by an increase.

**Study contributions**

**Main results**

Pesticide poisoning has increased, as well as case fatality ratio. The use of these products as a means of suicide attempt accounted for more than half of poisoning cases and practically all the deaths registered on the system.

**Implications for services**

The association between characteristics and case fatality ratio due to poisoning should be taken into consideration during the planning of prevention measures and control of poisonings. In addition, it is necessary to strengthen and integrate compulsory notification systems.

**Perspectives**

It is important to develop actions that can reduce the impact of pesticides on the health of the population and environment, such as the adoption of more sustainable agricultural production, as well as the improvement of surveillance and timely diagnosis actions.

## Introduction

Pesticides are used mainly in agriculture, although they can also be used in public health, veterinary medicine and the household environment, especially for insect and pest control.^1^ Inappropriate and abusive use of these substances has serious consequences for the environment and human health, such as deaths due to acute poisonings or diseases caused by chronic exposure,^2^ including infertility, impotence, abortion, congenital malformations, neurotoxicity, hormonal dysregulation, effects on the immune system and cancer.^3^

In Brazil, between 2015 and 2020, 716,912 cases of exogenous poisoning were registered on the Notifiable Health Conditions Information System (SINAN), of which 10.3% (73,835) were exposure to pesticides, including rodenticides and veterinary products. Regarding the cases of exposure to pesticides, 63.7% (456,602) were classified as confirmed poisoning,^4^ however, due to underreporting, it has been estimated that these percentages are even higher.^4^

Intensification of pesticide use, in Brazil, and the harm that exposure to these products causes to the population make monitoring the number of poisoning cases of fundamental importance for public health, as a way to help planning actions, define intervention priorities, in addition to enabling the evaluation of the impact of the proposed approaches.^5^

Although the cases of pesticide poisoning are compulsorily notifiable health conditions on SINAN, the analysis of the pattern of these occurrences in Brazilian municipalities is still limited.^5^ In Espírito Santo, a state with one of the highest incidences of pesticide poisoning per inhabitant,^6^ data on the subject are still scarce in the literature. Thus, this study aimed to describe the spatial distribution of cases of pesticide poisoning and analyze the temporal trend in the incidence rates and case fatality ratio due to this event in the state of Espírito Santo, Brazil.

## Methods

This was an epidemiological time-series study of confirmed cases of pesticide poisoning registered on SINAN, in Espírito Santo, between 2007 and 2016.

Espírito Santo is located in the Southeast region of Brazil and has 78 municipalities. According to the last population census of the Instituto Brasileiro de Geografia e Estatística (IBGE) available (2010), the state has a population of 3,514,952 inhabitants, an area of approximately 46,000 km^2^ and a human development index (HDI) of 0.740.^7^ Primary Health Care coverage for the state’s population is about 80%, while Family Health Strategy (ESF) coverage is 65%.^8^

In this study, we used records of notified poisoning cases in the state, made available by Espírito Santos State Department of Health (SESA/ES), through its Research Analysis Committee within the State Health Network. Data were generated through exogenous poisoning investigation forms input to SINAN by Municipal Health Departments, with data unification at state level and consolidation in the national database. In order to calculate the incidence rate and case fatality ratios of poisonings, information regarding the number of inhabitants of each municipality, made available by the Inter-Agency Health Information Network (RIPSA), was used.^9^

This study took into consideration the cases classified as 'confirmed poisonings' in the field 'final classification' of the SINAN form, in which the toxic agent responsible for poisoning had been registered as 'pesticide for agricultural use', 'domestic pesticide', 'pesticide/public health use', 'rodenticide' or 'veterinary product'. Records that did not have pesticides included in the list of substances that cause poisoning were excluded from the analysis, even though they fit one of the aforementioned classifications.

Incidence rate of poisoning cases was calculated taking into consideration the total number of registers of 'confirmed poisonings', according to the year and municipality where the notification was made, divided by the population estimates of each municipality for the respective year, multiplied by 100,000 inhabitants.

In order to determine the number of deaths, all cases classified as 'death due to exogenous poisoning' were taken into consideration in the field 'evolution of case'. Case fatality ratio in each municipality was calculated by dividing the total number of confirmed poisoning cases that progressed to death, by the total number of confirmed poisoning cases, multiplied by 100.

The variables studied, available in the individual notification form, were grouped into two categories: those related to sociodemographic characteristics and those related to exposure. Thus, the following sociodemographic variables were selected:

- toxic agent group (pesticide agricultural use; domestic use; for public health use; rodenticide; veterinary product);
- sex (female; male);
- age group (in years: <18, children and adolescents; ≥ 18 and < 60, adults; ≥ 60, older adults);
- race/skin color [White; Black; Brown; other (Asian or Indigenous); ignored];
- residential area (urban or peri-urban; rural); and
- situation in the labor market [with an employment relationship (public servant; formal job - work on the books; co-operative job; temporary job); without an employment relationship (casual employment; work off the books; self-employed; employer); unemployed; retired; others; ignored; no information provided].

For data related to pesticide exposure, we selected:

- place where the exposure occurred [residence; work environment or commuting to work; outside environment; other (health services; school/day care center; others); ignored; no information provided];
- exposure zone (urban or peri-urban; rural; ignored; no information provided);
- exposure/contamination circumstance [normal use; accidental; environmental; suicide attempt; other (day care centers; health services; outside environment; others); ignored; no information provided];
- type of exposure (acute single; acute repetitive; chronic or acute on top of chronic; ignored, no information provided);
- exposure route [digestive tract; skin; respiratory tract; other (ocular; parenteral; vaginal; transplacental; other), ignored; no information provided];
- (if) exposure resulting from work (yes; no; ignored; no information provided);
- agronomic classification of pesticides (fungicide; fungicide/insecticide; herbicide; insecticide; rodenticide; no information provided); and
- chemical group of pesticides (bipyridyl; carbamate; coumarin; N-substituted glycine; benzofuranyl methylcarbamate; organophosphate; pyrethroid; others; no information provided).

Agronomic classification of pesticides and chemical group were determined according to the name of the first toxic agent mentioned as cause of poisoning. The second or third toxic agent was taken into consideration only in cases, in which the substances mentioned in the first or second fields of the form, respectively, were not pesticides. The names that did not present exact correspondence were adjusted using nominal approximation, according to the list of pesticides of the Phytosanitary Pesticides System (AGROFIT)^10^ of the Ministry of Agriculture, Brazil’s Livestock and Food Supply (MAPA), or when we could not find them using electronic search engines. When it was not possible to identify the pesticide that caused poisoning, we took into consideration the agronomic classification mentioned in response to the question present in the notification questionnaire: *If it is a pesticide, what is the purpose of use?*

A descriptive analysis of the frequency distribution of sociodemographic and exposure of confirmed cases characteristics was performed by calculating absolute and percentage values, and case fatality ratios.

Temporal trends in the incidence rates and case fatality ratios of poisoning cases were projected based on the observation and analysis of scatter plots over the period. Overall trends were presented according to sex and age group of exposed/poisoned individuals. The association between case fatality ratios and sociodemographic and exposure characteristics was analyzed using Pearson's chi-square test and Fisher's exact test. Statistical significance of 5% was adopted.

For the construction of thematic maps of the distribution of poisoning cases, average incidence rates of cases per municipality of residence, standardized by age, according to the two quadrennial periods (2007-2011 and 2012-2016) and/or decade analyzed (2007-2016) were calculated. Statistical analyses were performed using the Statistical Package for the Social Sciences (SPSS) software, version 22. The choropleth maps were constructed using the QGIS 2.14 version.

The study project was approved by the Research Ethics Committee of the Center for Health Sciences (CEP/CCS/UFES) of Universidade Federal do Espírito Santo, Certificate of Submission for Ethical Appraisal (CAAE) No. 77009417.7.0000.5060, issued on September 26, 2017.

## Results

**Figure 1:**
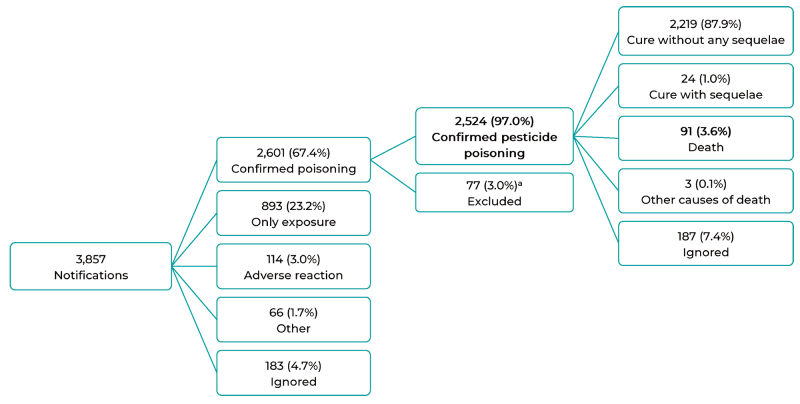
Flowchart of the final classification and outcome of cases of exogenous pesticide poisoning notified on the Notifiable Health Conditions Information System, Espírito Santo, Brazil, 2007-2016

A total of 3,857 cases of pesticide poisoning were notified on SINAN system, in the state of Espírito Santo, between 2007 and 2016. 2,601 (67.4%) of which were classified as 'confirmed poisoning' and of these, 77 (3.0%) were excluded from the study because they did not present pesticides among the substances that caused poisoning. Finally, a total of 2,524 poisoning cases were included in this analysis (Figure 1).

a) 77 individuals were excluded, despite being classified as confirmed exogenous poisoning cases, because they did not present pesticides among the possible substances that caused poisoning.

Regarding the characteristics of poisoning cases, they were most frequent in males (62.4%), adults (79.6%), among individuals of White race/skin color (37.9%) and those living in urban or peri-urban areas (52.3%) (Table 1).

**Table 1:** Frequency distribution of confirmed cases and case fatality ratios due to pesticide poisoning, according to sociodemographic characteristics, notified on the Notifiable Health Conditions Information System, Espírito Santo, Brazil, 2007-2016

Sociodemographic characteristics	Confirmed cases		Deaths (n)	Case fatality ratio (%)	p-value^b,c^
	n^a^	%			
**Sex**					
Female	950	37.6	29	3.3	0.262
Male	1,574	62.4	62	4.2	
**Age group**					
Child/adolescent	403	16.0	9	2.4	0.001^d^
Adult	2,009	79.6	70	3.8	
Older adult	112	4.4	12	11.7	
**Race/skin color**					
White	956	37.9	47	5.2	0.241^d^
Black	183	7.2	4	2.5	
Brown	893	35.4	31	3.6	
Other (Asian or Indigenous)	21	0.8	-	0.0	
Ignored	471	18.7	-	-	
**Situation in the labor market**					
With an employment relationship (public servant; formal job (work in the books); co-operative job; temporary job)	310	12.3	10	3.5	0.008^d^
Without an employment relationship (casual employment; work off the books; self-employed; employer)	710	28.1	25	3.7	
Unemployed	145	5.7	5	3.8	
Retired	62	2.5	9	15.5	
Others	352	13.9	12	3.7	
Ignored	713	28.3	-	-	
No information provided	232	9.2	-	-	
**Residencial area**					
Urban or peri-urban	1,285	52.3	30	2.6	< 0.001
Rural	1,172	47.7	60	5.4	

a) The indicator was calculated taking into consideration the total number of individuals whose information regarding the item 'evolution of case' was available (n = 2,337); b) Missing or ignored data were not considered for Pearson’s chi-square calculation; c) P-value related to the analysis of the association between exposure variables (sociodemographic characteristics) and case fatality ratios due to poisoning; d) Fisher's exact test.

With regard to confirmed cases, 91 (3.6%) died (Figure 1), with an average case fatality ratio of 3.6% during the study period. The highest case fatality ratio was found for the year 2007 (8.3%). Significantly higher case fatality ratios were found among older adults (p-value = 0.001), illiterate individuals (p-value = 0.035), retirees (p-value = 0.008) and individuals living in rural areas (p-value < 0.001). There was no statistical difference regarding case fatality ratio by gender and race/skin color (Table 1).

In relation to exposure characteristics, there was a predominance of poisoning by pesticide for agricultural use (60.3%), that occurred at home (62.3%), in urban or peri-urban areas (39.8%), resulting from suicide attempts (48.5%) (Table 2). There was a predominance of cases of poisoning due to acute-single exposure (80.1%), digestive tract (61.3%) and non-work-related exposure (63.0%). Cases of insecticide poisoning were the most frequent (31.6%). Regarding the chemical group, carbamate poisonings (22.4%) were the most common, although 23.9% of the cases did not mention this information.

**Table 2:** Frequency distribution of confirmed cases and case fatality ratios due to pesticide poisoning, according to exposure characteristics, based on the Notifiable Health Conditions Information System, Espírito Santo, Brazil, 2007-2016

Exposure characteristics	Confirmed cases		Deaths (n)	Case fatality ratio (%)	p-value^b,c^
	n^a^	%			
Toxic agent group					
Pesticide/agricultural use	1,523	60.3	72	5.0	0.005^d^
Pesticide/domestic use	220	8.7	2	0.9	
Pesticide/public health use	51	2.0	-	0.0	
Rodenticide	592	23.5	14	2.7	
Veterinary product	138	5.5	3	2.4	
**Place where exposure occured**					
Residence	1,573	62.3	64	4.4	0.001^d^
Work environment or commuting to work	616	24.4	8	1.3	
Outside environment	64	2.6	4	6.8	
Other (health services; school/daycare; other)	49	1.9	2	4.3	
Ignored	179	7.1	-	-	
No information provided	43	1.7	-	-	
**Exposure zone**					
Urban or peri-urban	1,003	39.8	26	2.8	0.009^d^
Rural	965	38.2	48	5.3	
Ignored	23	0.9	-	-	
No information provided	533	21.1	-	-	
**Contamination/exposure circumstances**					
Normal use	229	9.1	2	0.9	< 0.001
Accidental	695	27.5	4	0.6	
Environmental	169	6.7	0	0.0	
Suicide attempt	1,224	48.5	85	7.7	
Other (daycare; health services; outside environment; other)	156	6.2	-	0.0	
Ignored	40	1.6	-	-	
No information provided	11	0.4	-	-	
**Type of exposure**					
Acute-single	2,022	80.1	72	3.8	0.043^d^
Acute-repetitive	175	6.9	4	2.5	
Chronic or acute on top of chronic	22	0.9	3	15.0	
Ignored	234	9.3	-	-	
No information provided	71	2.8	-	-	
**Exposure route**					
Digestive tract	1,547	61.3	82	5.9	< 0.001^d^
Skin	226	9.0	1	0.5	
Respiratory tract	626	24.8	3	0.5	
Other (ocular; parenteral; vaginal; transplacental; other)	29	1.1	-	0.0	
Ignored	7	0.3	-	-	
No information provided	89	3.5	-	-	
**Exposure resulting from work**					
Yes	744	29.5	4	0.6	< 0.001
No	1,591	63.0	77	5.3	
Ignored	132	5.2	-	-	
No information provided	57	2.3	-	-	
**Agronomic classification of pesticides**					
Fungicide	88	3.5	4	4.7	0.127^d^
Fungicide/insecticide	36	1.4	-	0.0	
Herbicide	675	26.8	35	5.5	
Insecticide	798	31.6	28	3.7	
Rodenticide	674	26.7	17	2.9	
No information provided	253	10.0	-	-	
**Chemical group of pesticides**					
Bipyridyl	66	2.6	18	34.6	< 0.001^d^
Carbamate	565	22.4	16	3.2	
Coumarin	56	2.2	-	0.0	
N-substituted glycine	431	17.1	5	1.2	
Benzofuranyl methylcarbamate	150	5.9	4	2.7	
Organophosphate	94	3.7	4	4.4	
Pyrethroid	161	6.4	1	0.6	
Other	399	15.8	-	-	
No information provided	602	23.9	-	-	

a) The indicator was calculated taking into consideration the total number of individuals whose information regarding the item 'evolution of case' was available (n = 2,337); b) Missing or ignored data were not considered for Pearson’s chi-square calculation; c) P-value related to the analysis of the association between exposure variables (sociodemographic characteristics) and case fatality ratios due to poisoning; d) Fisher's exact test.

The variables related to pesticide exposure were statistically associated with case fatality ratios, with the exception of the variable 'agronomic classification of pesticides'. Exposures that presented the highest case fatality ratios were those that occurred in the external environment (6.8%), in rural areas (5.3%), and those resulting from suicide attempts (7.7%). The exposures 'chronic or acute on top of chronic' (15.0%; p-value = 0.043) and 'digestive tract' (5.9%; p-value = < 0.001) were associated with the highest case fatality ratios. In relation to the association of case fatality ratio and chemical groups, exposure to bipyridyls, organophosphates and carbamates, corresponding to 34.6%, 4.4% and 3.2% of case fatality ratio, respectively, had the highest indicators (Table 2).

**Figure 2:**
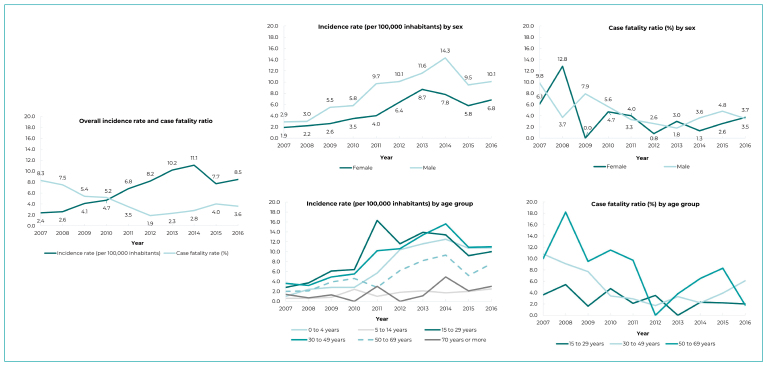
Incidence rate per 100,000 inhabitants and case fatality ratio by sex and age group of pesticide poisoning notified on the Notifiable Health Conditions Information System, Espírito Santo, Brazil, 2007-2016

The incidence rates of poisonings showed an increasing trend for the total number of cases. There was a small decrease in 2015 (7.7%), with a subsequent increase in the following year (Figure 2). There was an increase in the incidence rate of poisonings when the pattern by sex was analyzed: there was an increase in the trend corresponding to both males and females, in the period (Figure 2). The same pattern was observed when analyzing the evolution of the incidence rates of poisoning by age group, except for individuals aged 70 years and older, for whom the trend remained stable throughout the study period (Figure 2).

With regard to case fatality ratios, they had decreased until 2012, when they began to present an increased trend until the end of the historical series, in 2016 (Figure 2). There was no difference in the pattern of case fatality ratios between females and males over the period 2007-2016. Regarding case fatality ratios by age group, the differences were significant only among individuals aged 30 to 40 and 50 to 69.

**Figure 3:**
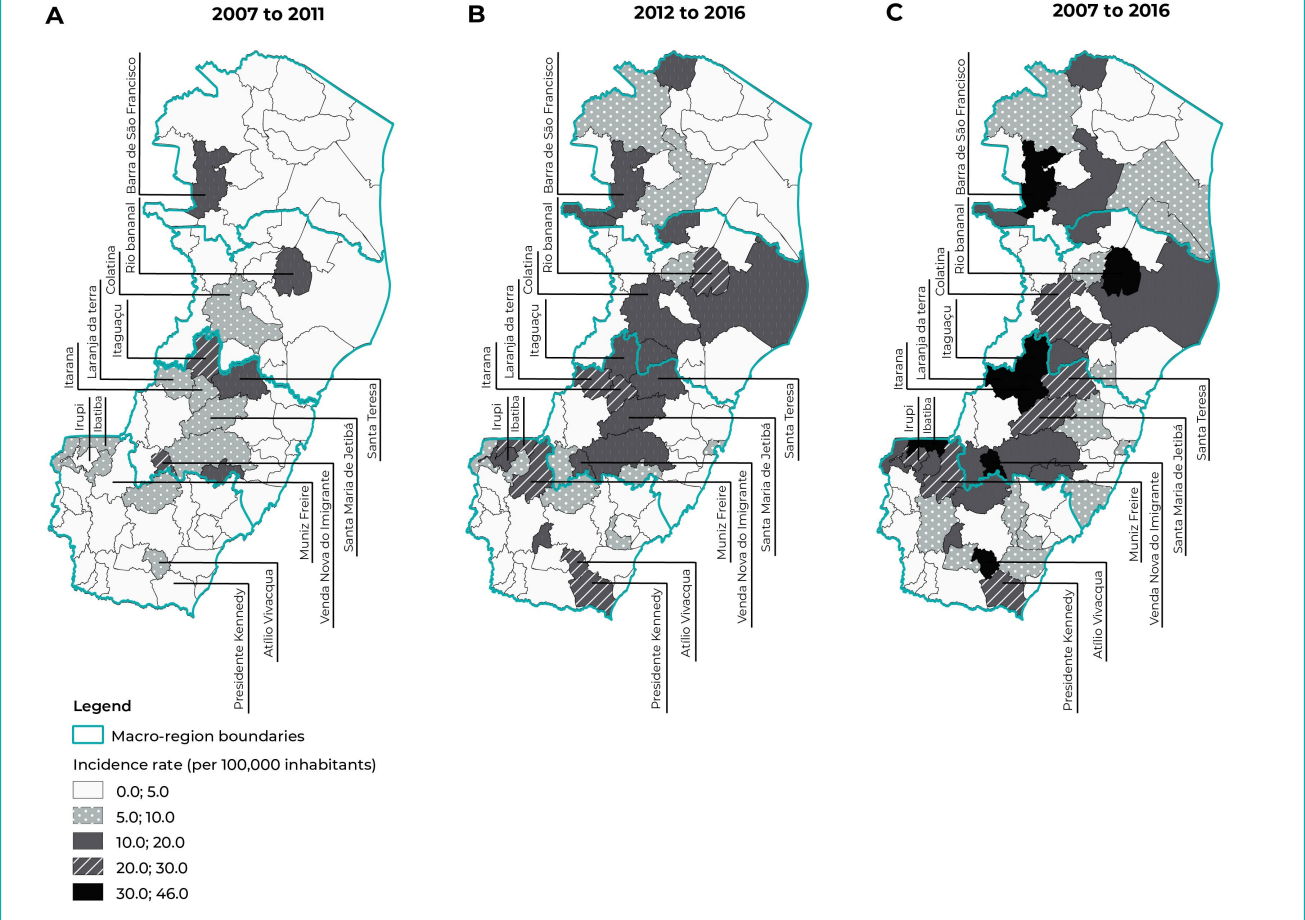
Average incidence rates of pesticide poisoning, Espírito Santo, Brazil, 2007-2011, 2012-2016 and 2007-2016

In relation to spatial distribution of poisoning cases, the highest incidences rates (30 to 46 new cases/100,000 inhabitants) were found for the municipalities of Barra de São Francisco, Rio Bananal, Itaguaçu, Laranja da Terra, Itarana, Venda Nova do Imigrante, Ibatiba and Atílio Vivácqua. In Colatina, Santa Teresa, Santa Maria de Jetibá, Irupi, Muniz Freire and Presidente Kennedy, the incidence rate calculated was 20 to 30 cases per 100,000 inhabitants. (Figure 3).

## Discussion

The incidence rates of confirmed pesticide poisonings in the state of Espírito Santo showed an upward temporal trend between 2007 and 2016. Case fatality ratios, however, showed a downward trend until 2012, and then presented an increase again at the end of the historical series. Sociodemographic factors, such as older age, being retired and living in rural areas, showed a positive association with the highest case fatality ratio of poisonings, as well as factors related to exposure and the chemical group of pesticides used.

In the present study, poisoning cases registered between 2007 and 2016 corresponded to 9.6% of the total number of notifications registered in the country in the same period. Most of these cases evolved to cure without sequelae, and case fatality ratio was similar to the national average (4.6%) and the average in the Southeast region (3.7%),^4^ taking into consideration the same period analyzed. When comparing this investigation data with the number of deaths registered on the Mortality Information System (SIM), it can be seen significant underreporting in the SINAN data. While 7,641 deaths due to pesticide poisoning were registered on SIM, in the country, of which 136 in Espírito Santo,^11^ on SINAN, only 3,193 and 91 were registered, respectively. Although the highest number of notifications of deaths due to poisoning was registered on the SIM system, in this article we have chosen to use SINAN data in order to evaluate the incidence rates and spatial distribution of poisoning cases, rather than only deaths registered on the system.

Notwithstanding there was a higher frequency of notification among males and individuals of White race/skin color, there was no significant difference in case fatality ratio in relation to these two variables, disagreeing with Bochner et al.,^12^ authors who identified a higher case fatality ratio among males when evaluating deaths due to poisoning occurred in Brazil from 2010 to 2015, based on SIM data. When analyzing the age distribution of cases, the group with the highest number of records was comprised of economically active population, consistent with the notification profile found in Rio Grande do Sul^13^ and Pernambuco.^14^ In addition to expenditure on health treatments, poisoning in working age population has one more aggravating factor: financial impacts related to the reduction of work capacity of these individuals and absenteeism, either due to death or physical or psychological limitations arising from exposure to pesticides.^15^

Although the highest number of poisonings was found in adult individuals, case fatality ratio was higher among older adults. It is common knowledge that during aging, there is a decrease in the biochemical capacity of metabolization of xenobiotic substances, by reducing enzymes responsible for the biotransformation of compounds, such as the cytochrome P-450s system, which increases the vulnerability of this age group.^16^ In addition, the large number of deaths among older adults may also be associated with the highest rates of suicide due to the ingestion of pesticides observed among them.^17^

Regarding the type of exposure, the most frequent occurrence of acute cases notified on the information systems can be attributed to the sudden and prominent onset of symptoms, in these cases, resulting in seeking emergency health services.^18^ Moreover, chronic poisonings are under-reported, given the difficulty in establishing a relationship between exposure and effect, especially when the clinical picture is undefined, unspecific, subtle and takes a long time.^7^ It is noteworthy that case fatality ratio was higher in cases related to types of exposure 'chronic or acute on top of chronic', reinforcing the harmful potential and irreversible damage that chronic exposure to these products causes to health.^2^ It is worth mentioning that the limits of pesticide residue present in food and water in Brazil exceed, in some cases, a value 1,800 times higher than that found for the European Union.^19^ This is another fact that can interfere in chronic exposure to pesticides and, consequently, in its effects on the health of the Brazilian population in the long term.

Approximately half of the exogenous poisonings and practically all deaths registered on the system were due to the use of pesticides as a means of suicide attempts. The ease of access to these products is a factor that directly contributes to these results.^20^ As an aggravating factor, many pesticides are neurotoxic, can lead to the emergence of depression and, consequently, suicidal ideation.^21^ It is estimated that banning highly hazardous pesticides in the 14 countries studied by Lee et al.^21^ could prevent about 28,000 suicide deaths each year.

Occupational exposure accounted for 744 people who were poisoned in Espírito Santo, in the period analyzed, which corresponds to about 1/3 of the notifications. Nevertheless, case fatality ratio was approximately nine times lower when compared to non-occupational exposure. However, the results need to be evaluated with caution, given that the difficulty in measuring chronic exposures, such as those that occur in the work environment, may lead to underreporting of occupational poisonings. The same reasoning applies to the evaluation of case fatality ratio of this type of exposure, given that the effects on health are not as easy to measure as those resulting from acute exposure present in suicide attempts, for example.^22^ Regarding the agronomic classification of pesticides, insecticides were the most frequently reported pesticide among the cases studied, followed by rodenticides and herbicides. It is worth highlighting that in this study, 548 (21.7%) occurrences of poisoning were attributed to the rodenticide known as "chumbinho" (data not shown). This product belongs to the chemical group of carbamates and it is commonly used in suicide attempts. Despite being banned in Brazil, it has been sold illegally,^23^ reinforcing the need for a stricter control of the marketing of this product.

The highest case fatality ratio in the period studied was associated with the chemical group of bipyridyls, found in paraquat-based herbicides. This compound is frequently used in suicide attempts and its highly toxic action results from its ability to produce free radicals continuously and lipid peroxidation in cell membranes.^24^

The increase in pesticide poisoning in Espírito Santo follows the trend found for other regions of the country. In the same period analyzed, there was an increase of 116% confirmed poisoning cases in Brazil, and 194% in the Southeast region specifically.^4^ The increase in the marketing of these substances in the period^25^ possibly contributed to the increase in the number of poisonings registered in the system. That is, the more the country relaxes the authorization of use and the marketing of these products, the greater the incidence rate of poisoning in the population.^26^ In addition, the presence of pesticides with active ingredients of high acute toxicity, many of them have already been banned in the European Community and the United States, exposes the worker to an increasing risk of poisoning.^27^ It is worth highlighting that the best practices of Health Surveillance in Populations Exposed to Pesticides may also have contributed to the increase in the capture of poisoning data by notification systems.^1^

The decline in case fatality ratio in the period prior to 2012 may have occurred due to the reduction in the number of deaths among incident cases, which increased during the historical series, or even due to the possibility of underreporting in this period. The declining trend in mortality due to pesticides was also identified by Bochner and Freire^12^ when evaluating SIM data between 2010 and 2015.

Incidence rates of poisoning greater than 20 per 100,000 inhabitants were identified in several municipalities in Espírito Santo, including those in the mountainous region of the state. Similar results were identified by Bombardi,^19^ whose research presented four of the five municipalities in Espírito Santo studied, included in the same mountainous region, that also showed a higher number of cases of poisoning by pesticides for agricultural use, in relation to the municipal population. There are significant agricultural activities in these areas, therefore it is possible that frequent contact with pesticides have contributed directly to the results found.

This study has limitations such as (i) the possibility of information being filled out incorrectly on data collection forms, (ii) the large amount of information reported as ignored or that had not been filled in and (iii) the possible occurrence of underreporting, especially regarding chronic poisonings, given limited knowledge and difficulty in diagnosing. Underreporting may also be related to the difficulty of access to health services faced by users, especially in rural areas. The lack of knowledge of health professionals regarding the diagnosis, the banalization of exposure and the fear of possible employer’s retaliation, may also compromise the number of notifications registered.^28^

Despite these limitations, trend studies are important to understand the evolution of this disease, showing the morbidity pattern in the period studied, and the profile of the affected population. These data are an important source of information in order to guide and direct the development and planning of government actions and interventions necessary to prevent poisonings in the state.

Taking these results, it can be concluded that the number of pesticide poisonings in Espírito Santo has increased, as well as case fatality ratio, and that the use of pesticides as a means of suicide attempt accounted for more than half of poisoning cases and practically all of the deaths registered on the system. In addition, it could be seen that sociodemographic and exposure characteristics have been associated with deaths due to poisoning, a fact that should be taken into consideration during the planning of actions aimed at prevention and control of poisonings. These data reinforce the difficulties that the increasing exposure to pesticides causes to the population. They also highlight the need for actions, not only actions coming from the health sector, but also those coming from others, which have a direct relationship with the use of pesticides in the country, as a way to reduce the impacts of these products on the health of the population and environment. Among the possible measures to be taken, we highlight the strengthening and integration of compulsory notification systems as a way to qualify database, improving the completeness and consistency of information. In addition, measures involving (i) health team training aiming at promoting surveillance actions and timely diagnosis of poisoning cases, (ii) the interconnection of health care networks, (iii) health education for the most vulnerable populations and (iv) strengthening public policies aimed at these individuals, including stricter control of records, marketing and use of pesticides.

Adoption of a more sustainable agricultural production, such as agroecology, can be a way to cope with this scenario. Furthermore, longitudinal studies can contribute to evaluate chronic exposure to pesticides and their relationship with poisonings and chronic diseases, as well as a further discussion on the social determinants of pesticide use, that is also necessary.
